# Elevated FOXO6 expression correlates with progression and prognosis in gastric cancer

**DOI:** 10.18632/oncotarget.15920

**Published:** 2017-03-06

**Authors:** Jia-Hong Wang, Hong-sheng Tang, Xiao-Shan Li, Xiang-Liang Zhang, Xian-Zi Yang, Li-Si Zeng, Qiang Ruan, Yong-Hong Huang, Gao-Jie Liu, Jin Wang, Shu-Zhong Cui

**Affiliations:** ^1^ Department of Abdominal Surgery, Affiliated Cancer Hospital & Institute of Guangzhou Medical University, Guangzhou, 510095, Guangdong, China; ^2^ Department of Medical Oncology, Affiliated Cancer Hospital & Institute of Guangzhou Medical University, Guangzhou, 510095, Guangdong, China

**Keywords:** FOXO6, gastric cancer, aggressiveness, prognosis

## Abstract

The FOXO6 correlated with tumor progression in a wide range of carcinomas, yet little is known in gastric cancer. The expression of FOXO6 and matrix metallopeptidase 9 (MMP-9) was assessed by immunohistochemistry in 192 gastric carcinoma specimens. The correlation between FOXO6 expression with MMP-9, clinicopathological/prognostic value in gastric cancer was examined. FOXO6 overexpression was significantly associated with depth of invasion, lymph node metastasis and stage of disease. In univariate and multivariate analyses, FOXO6 was an independent prognostic factor for both overall survival (OS) and recurrence-free survival (RFS). Moreover, FOXO6 over-expression was correlated with poor prognosis in patients subgroups stratified by tumor size, depth of invasion and lymph node metastasis. FOXO6 expression was increased in both prominent serosal invasion group and lymph node metastasis group. In addition, FOXO6 expression was positively correlated with MMP-9 among 192 gastric cancer tissues. Patients with FOXO6 over-expression had poor OS and shorter RFS in low and high invasiveness groups. Furthermore, stratified analysis showed that the TNM stage I patients with high FOXO6 expression had poor prognosis than those with low FOXO6 expression. In conclusion, FOXO6 overexpression promotes tumor aggressiveness and prognosis, and could be a promising target for prognostic prediction in gastric cancer patients.

**Condensed abstract:**

The aim of this study was to analyze the role of FOXO6 in patients with gastric carcinoma. FOXO6 may play an important role on tumor invasion, metastasis and prognosis. It may also serve as a novel target for prognostic prediction.

## INTRODUCTION

Gastric cancer is of great threat to human worldwide due to its high incidence and mortality among cancers [1–4]. The incidence of gastric cancer in China and some East Asia countries is much higher than that in America or Europe countries [5, 6]. Surgery is the preferred method for the treatment of gastric cancer. Meanwhile, platinum-based adjuvant chemotherapy after surgery has been widely accepted as a standard treatment for several decades. However, due to atypical symptoms at the early stage, over 80% of patients with gastric cancer were diagnosed at an advanced stage, which usually indicates a poor prognosis [7]. Moreover, chemotherapy has limited efficacy in both resectable and unresectable gastric cancer cases [8, 9]. Different kinds of biomarkers are found which are correlated with different types of cancer [10]. Therefore, new molecular markers, which are pivotal to tumor biology, to prediction of the prognosis and adjuvant treatment regimens, are urgently needed [11–14].

Forkhead box (FOXO) gene family is evolutionarily conserved in the human genome with a similar sequence among its members and most of its mediated reactions are related to the insulin/PI3K/AKT signaling pathway [15]. The presently found FOXO gene families in mammal include FOXO1, FOXO3, FOXO4 and FOXO6. It has been reported that FOXO6 played an important role in oxidative stress of cell proliferation [16, 17]. Moreover, previous datas revealed that the FOXO6 could regulate the synaptic function and hepatic glucose homeostasis in mice [18, 19], and promote oncogenicity of gastric cancer via upregulation of C-myc signal pathway [20]. However, the prognosis of FOXO6 gene in gastric cancer remains unknown. Therefore, the purpose of this study is to investigate the correlation of FOXO6 expression with clinicopathological features and prognosis in gastric cancer.

## RESULTS

### Correlation of FOXO6 expression with clinicopathological features

**Figure 1:**
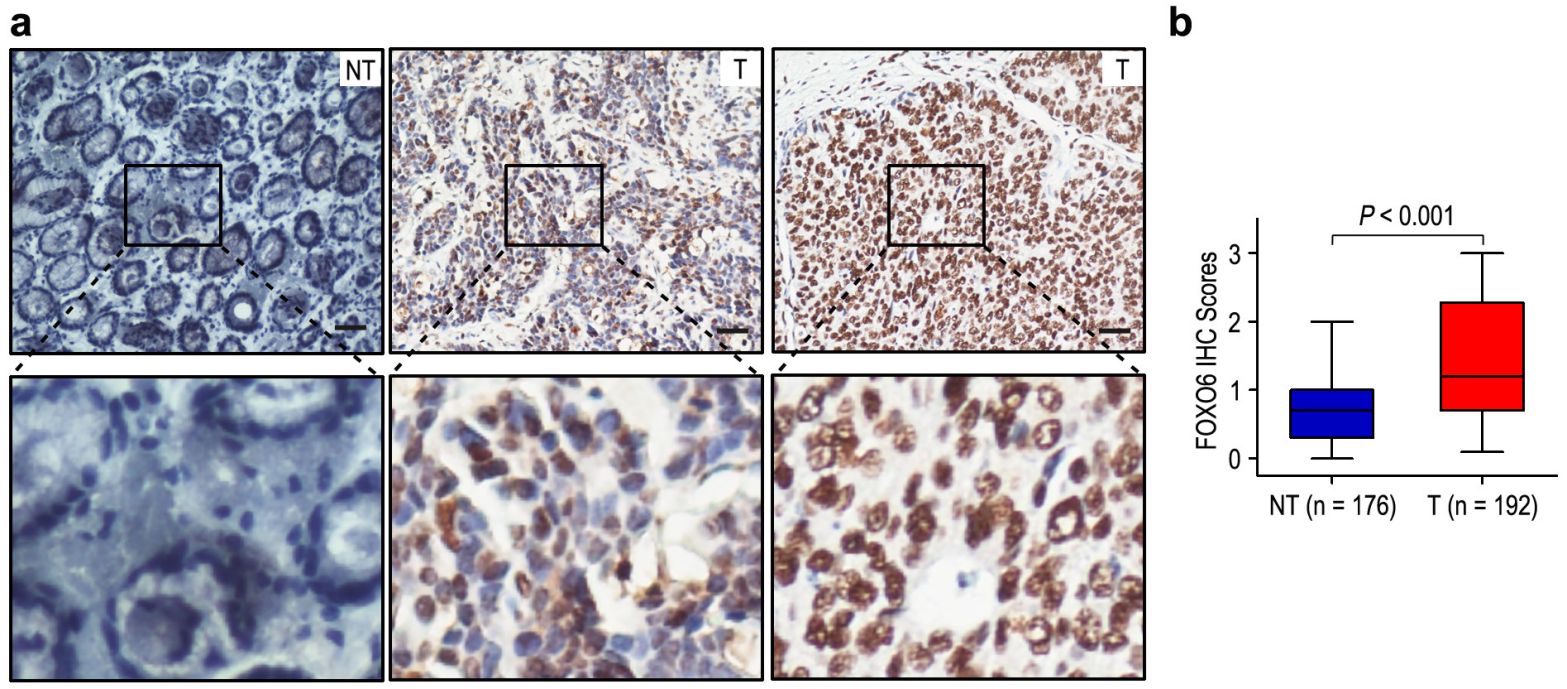
FOXO6 was significantly over-expression in gastric cancer **a**. IHC assays of FOXO6 expression in 192 paired gastric cancer samples and adjacent non-tumorous tissues. The upper left panel represents low FOXO6 expression in adjacent non-tumorous tissues. The upper middle and right panel represents low and high FOXO6 expression in gastric cancer. Lower panels represent magnified pictures of boxed area in the corresponding upper panels. The scale bar represents 50 μm. **b**. FOXO6 expression levels were compared with gastric cancer and adjacent non-tumorous specimens. Statistical analysis was performed by Paired-Samples *t*-test.

We firstly detected FOXO6 expression in 192 gastric cancer specimens, as compared with the levels in matched adjacent non-tumorous gastric tissues. The expression of FOXO6 was mainly in the nucleus of gastric cancer cells (Figure 1a). High FOXO6 expression was found in 98 of the 192 (51.0%) gastric cancer samples, compared with 31/176 (17.6%) in para-carcinoma tissues (*P* < 0.001; Figure 1b). Our results suggested that FOXO6 was overexpression in gastric cancer tissues.

To verify the functions of FOXO6 in gastric cancer, we correlated FOXO6 expression with other widely recognized clinicopathologic features (Table 1). Overexpression of FOXO6 was positively related to depth of invasion, lymph node metastasis and stage of disease in gastric cancer (all *P* < 0.001) (Table 1).

**Table 1:** Clinicopathologic correlation of FOXO6 expression in 192 gastric cancer patients

Characteristics	No. of patients	FOXO6 expression (%)		*P*-value
		Low	High	
Gender				
Male	122	55 (45.1%)	67 (54.9%)	
Female	70	39 (55.7%)	31 (44.3%)	0.156
Age (years)				
≤ 60	123	57 (46.3%)	66 (53.7%)	
> 60	69	37 (53.6%)	32 (46.4%)	0.333
Size (cm)				
≤ 5.0	126	63 (50.0%)	63 (50.0%)	
> 5.0	66	31 (47.0%)	35 (53.0%)	0.690
Tumor site				
Upper	81	39 (48.1%)	42 (51.9%)	
Middle/Lower	111	55 (49.5%)	56 (50.5%)	0.848
Differentiation				
Well/Moderate	88	37 (42.0%)	51 (58.0%)	
Poor	104	57 (54.8%)	47 (45.2%)	0.078
Depth of invasion				
T1/T2	74	48 (64.9%)	26 (35.1%)	
T3/T4	118	46 (39.0%)	72 (61.0%)	< 0.001
Lymph node metastasis				
Negative	52	37 (71.2%)	15 (28.8%)	
Positive	140	57 (40.7%)	83 (59.3%)	< 0.001
Stages				
I/II	93	59 (63.4%)	34 (36.6%)	
III	99	35 (35.4%)	64 (64.6%)	< 0.001

### Overexpression of FOXO6 correlated with poor prognosis in gastric cancer

Moreover, to confirm the value of FOXO6 expression on survival (OS and RFS) in patients with gastric cancer, we analyzed the correlation between clinicopathologic parameters and patients outcomes by univariate analysis. The results revealed that tumor size, depth of invasion, lymph node metastasis and FOXO6 status (all *P* < 0.05) were independent factors that affected OS. While larger tumor size, upper tumor site, prominent serosal invasion, lymph node metastasis and FOXO6 overexpression (all *P* < 0.05) were unfavourable predictors for RFS (Table 2). In addition, FOXO6 expression and the prognostic parameters found by univariate analysis were entered into a multivariate model to identify the independent predictors of OS and RFS. Our data revealed that FOXO6 overexpression was a negatively independent predictor for OS in patients with gastric cancer (HR = 3.275, 95% CI: 2.201-4.873; *P* < 0.001). Furthermore, the patients with FOXO6 overexpression were more likely to suffer from recurrence than that with low FOXO6 expression (HR = 3.077, 95% CI: 2.089-4.532; *P* < 0.001) (Table 2).

**Table 2:** Univariate and multivariate analysis of FOXO6 associated with survival and recurrence in gastric cancer patients

Variables*	OS				RFS			
	Univariate		Multivariate		Univariate		Multivariate	
	*P*-value	*P*-value	HR	95% CI	*P*-value	*P*-value	HR	95% CI
Gender (Female vs. Male)	NS	NS			NS	NS		
Age, years (≤ 60 vs. > 60)	NS	NS			NS	NS		
Tumor size (cm) (≤ 5.0 vs. > 5.0)	0.001	0.031	1.479	1.037-2.110	0.001	0.025	1.508	1.052-2.162
Tumor site (Upper vs. Middle/Lower)	NS	NS			0.050	NS		
Tumor differentiation (Well/Moderate vs. Poor)	NS	NS			NS	NS		
Depth of invasion (T1/T2 vs. T3/T4)	< 0.001	0.001	2.468	1.418-4.293	< 0.001	0.001	2.449	1.431-4.191
Lymph node metastasis (Negative vs. Positive)	< 0.001	0.018	2.332	1.158-4.693	< 0.001	0.007	2.567	1.299-5.074
FOXO6 (Low vs. High)	< 0.001	< 0.001	3.275	2.201-4.873	< 0.001	< 0.001	3.077	2.089-4.532

*TNM stage was combined with depth of invasion and lymph node metastasis; we did not enter the TNM stage into multiple analysis with these indexes to avoid any bias in analysis.

Abbreviations: OS overall survival, RFS recurrence-free survival, NS not significant, HR hazard ratio, CI confidential interval.

**Figure 2:**
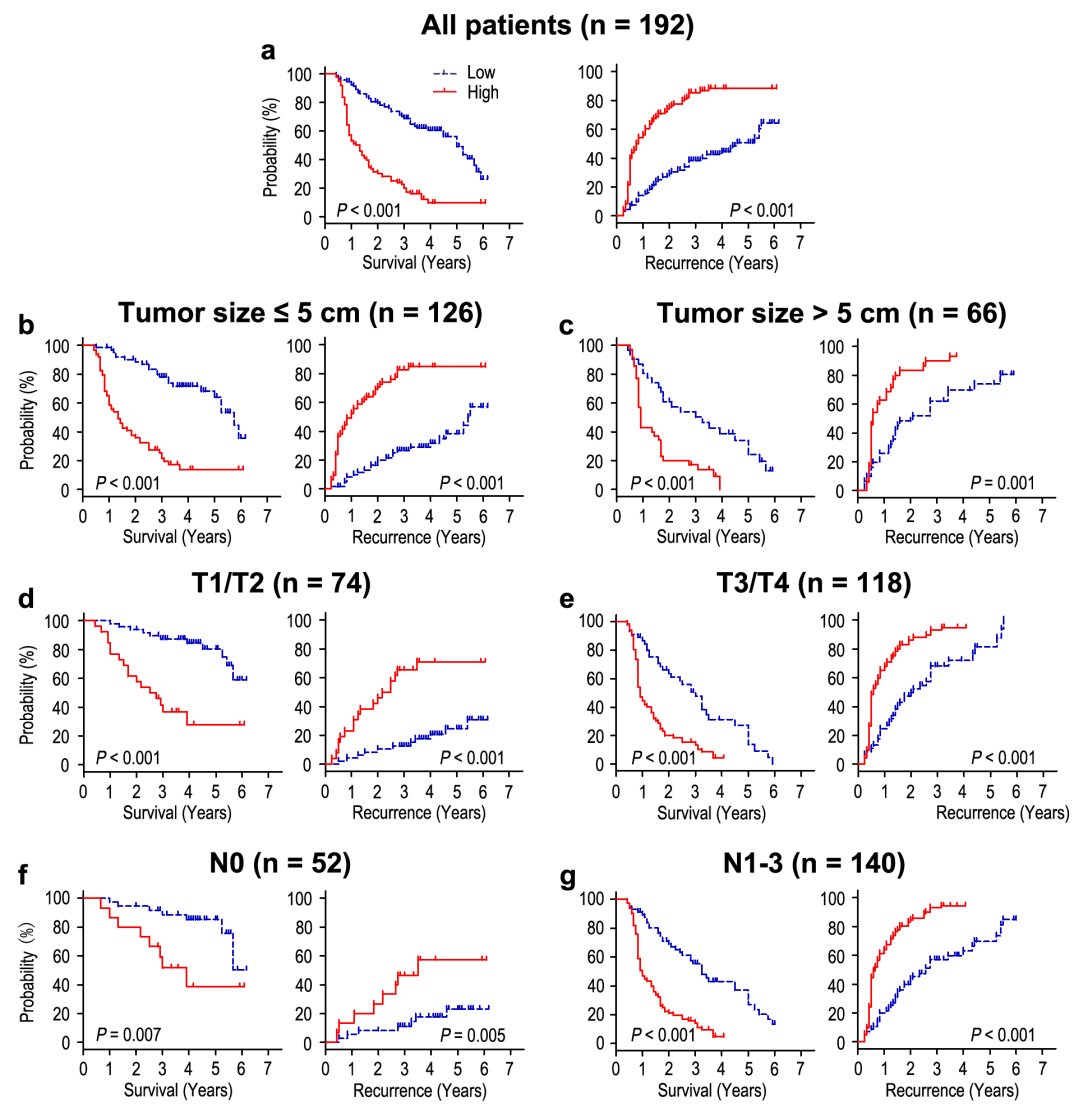
Overall survival and Recurrence-free survival are shown for gastric cancer patients All patients were stratified according to tumor size, depth of invasion and lymph node metastasis. Kaplan-Meier survival estimates and log-rank tests were used to analyze the prognosis of FOXO6 expression in all patients **a**. and each subgroup **b-g**.

The prognosis analysis showed that the patients with FOXO6 overexpression had poorer OS and RFS than that with low FOXO6 expression (both *P* < 0.001) (Figure 2a). The median times of OS and RFS were 25.5 months and 19.5 months in all the gastric cancer patients. Moreover, the median times of OS and RFS in FOXO6 over-expression (n = 98) gastric cancer patients were 15.0 months and 9.5 months, which were significantly shorter than those with FOXO6 low-expression (n = 94) (36.0 months and 39.0 months). In addition, the 5-year OS and RFS rates of the FOXO6 highly expression were significantly lower (9.6% and 11.6%), compared with that of FOXO6 low-expression (56.2% and 49.3%) (Figure 2a). Furthermore, in order to assess the significance of FOXO6 in prognosis, all the patients were divided into different subgroups basing on tumor size, depth of invasion, and lymph node metastasis (Figure 2b-2g). Our results showed that FOXO6 overexpression could keep its prognostic value in predicting poorer survival (OS and RFS) in different subgroups (all *P* < 0.05). Therefore, it suggests that FOXO6 could be a potential prognostic biomarker for different risk of gastric cancer patients.

### FOXO6 overexpression predicts poor prognosis independent of tumor invasiveness

**Figure 3:**
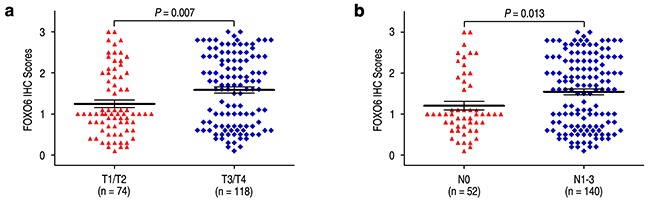
Comparsion of FOXO6 expression by depth of invasion and lymph node metastasis FOXO6 expression is markedly increased both prominent serosal invasion group **a**. and lymph node metastasis group **b**.

**Figure 4:**
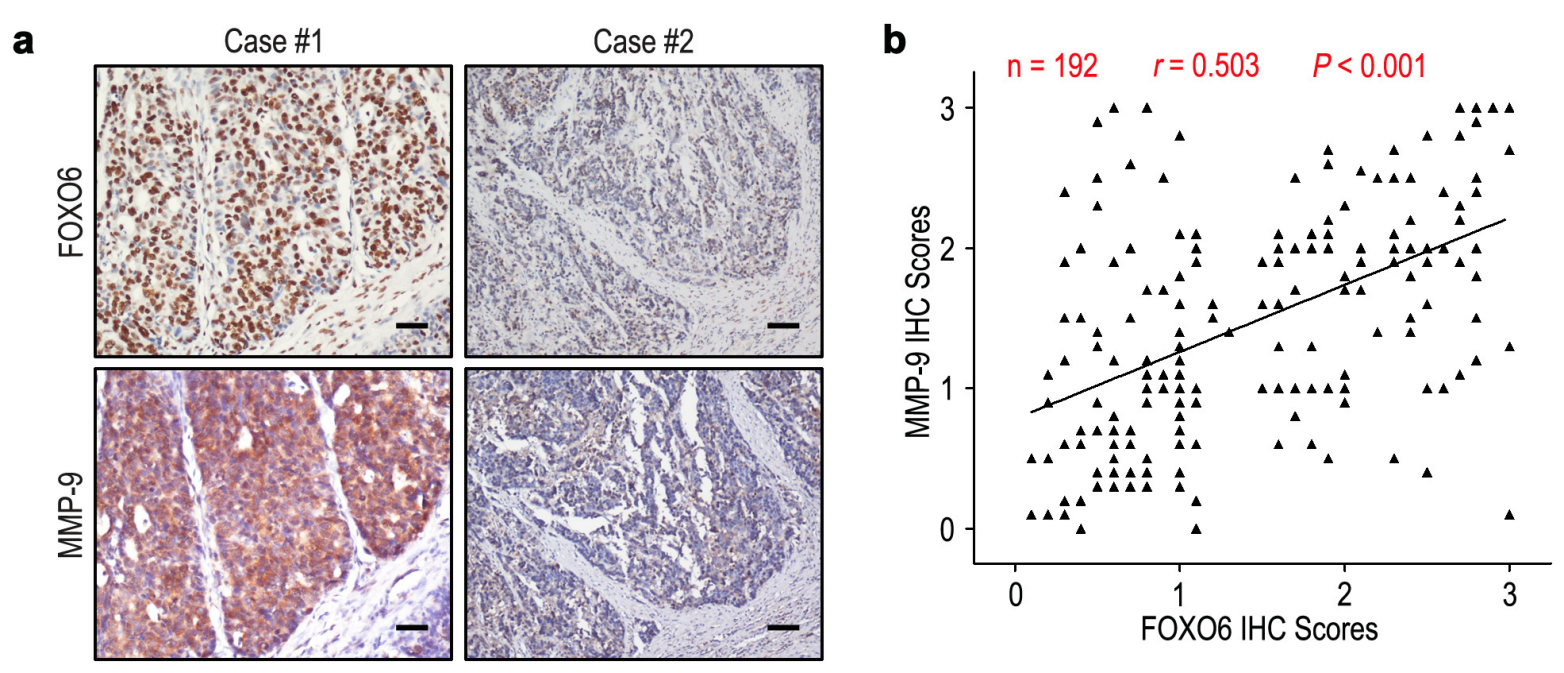
FOXO6 and MMP-9 levels correlated in 192 gastric cancer tissues **a**. Serial sections of gastric cancer tissues were subjected to IHC staining with antibodies against FOXO6 and MMP-9. In case #1, high expression of FOXO6 in gastric cancer tissues was accompanied by elevated MMP-9. In case #2, low expression of FOXO6 was accompanied by the absence of MMP-9. The scale bar represents 50 μm. **b**. Spearman correlation analysis between FOXO6 and MMP-9 expression in 192 gastric cancer patients by IHC assays. FOXO6 expression was positively correlated with MMP-9 expression.

We evaluated the relationship of FOXO6 expression with depth of invasion and lymph node metastasis in gastric cancer. FOXO6 expression was higher in the groups of T3/T4 and N1-3, compared with those in the groups of T1/T2 and N0 (*P* = 0.007 and *P* = 0.013, respectively) (Figure 3). Previous study found that MMP-9 expression was associated with aggressiveness in gastric cancer. Therefore, the correlation of FOXO6 and MMP-9 expression was evaluated in gastric cancer patients. The relationship of FOXO6 and MMP-9 expression was verified by IHC methods in serial sections of gastric cancer samples (Figure 4a). Our data revealed that FOXO6 was significantly associated with MMP-9 expression in 192 gastric cancer samples (*r* = 0.503, *P* < 0.001, Figure 4b).

**Figure 5:**
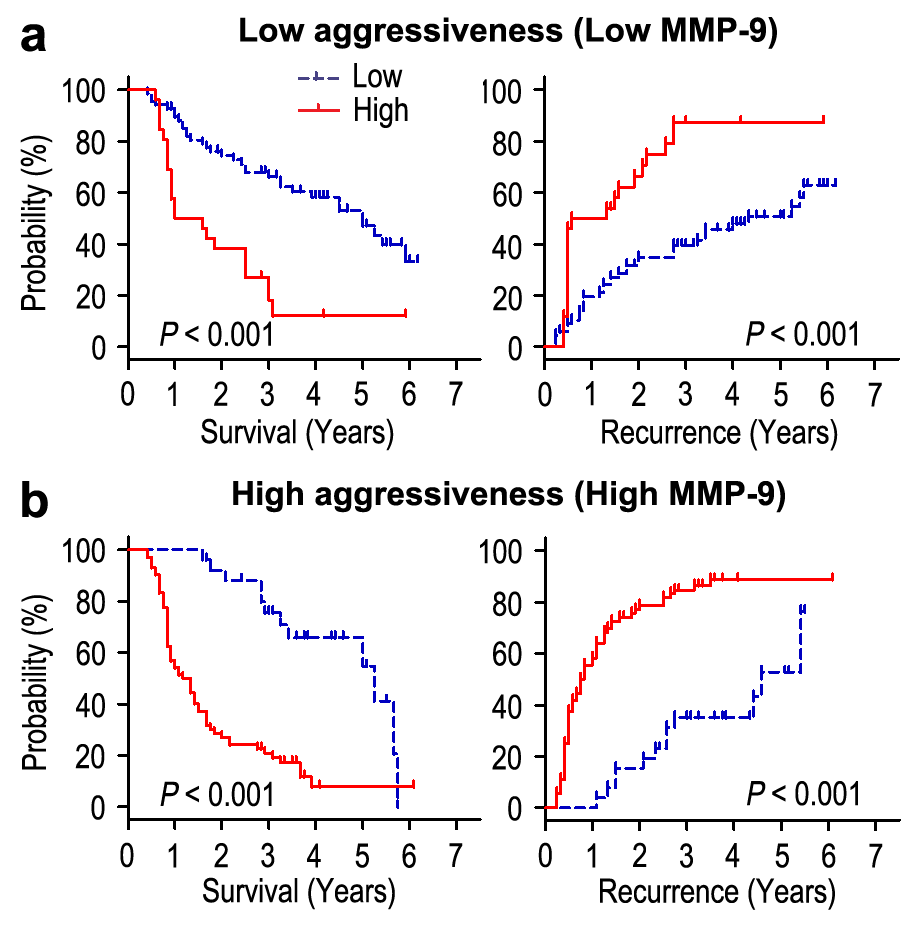
Overall survival and Recurrence-free survival are shown for patients with low tumor invasiveness **a**. and highly invasiveness **b**. Kaplan-Meier survival estimates and log-rank tests were used to analyze the association between FOXO6 expression and overall survival or time to recurrence in patients with low invasiveness (low MMP-9; n = 94) or high invasiveness (high MMP-9; n = 98).

Moreover, we evaluated the effect of invasiveness on the prognosis of FOXO6 expression in the way of taking MMP-9 as a marker for invasive potential of cancer cells. The patients of gastric cancer were stratified either minimal-invasive group (MMP-9 low-expression; n = 94) or highly invasive group (MMP-9 over-expression; n = 98) according to the expression of MMP-9 potein. The correlation of FOXO6 expression and survival was analyzed by Kaplan-Meier survival curves in different invasive potential of patients. In the minimal-invasive group, the patients with FOXO6 overexpression were related to poorer OS (*P* < 0.001) and shorter RFS (*P* < 0.001), compared with those with FOXO6 low-expression (Figure 5a). In the highly invasive group, patients with FOXO6 overexpression were prone to death and tumor relapse (both *P* < 0.001) (Figure 5b). Therefore, FOXO6 could be a potential prognostic indicator for gastric cancer patients that independent of tumor invasion.

### Prognostic value of FOXO6 in early gastric cancer patients

**Figure 6:**
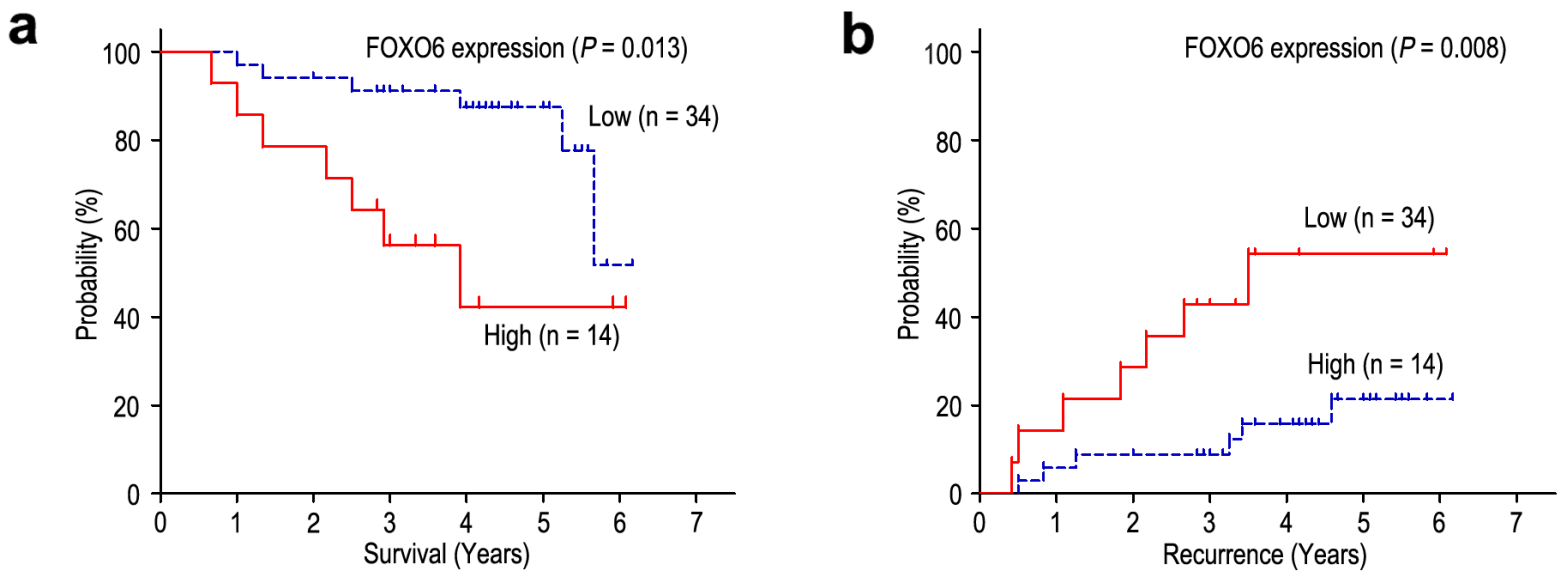
FOXO6 overexpression indicates poor prognosis in TNM stage I gastric cancer patients Overall survival **a**. and Recurrence-free survival **b**. curves were generated based on the FOXO6 protein expression statuses in 48 TNM stage I patients.

We further investigated the prognostic value of FOXO6 expression in TNM stage I patients. For the 48 TNM stage I patients, significant correlations were found between FOXO6 expression and OS (*P* = 0.013) and RFS (*P* = 0.008) (Figure 6). In the multivariate model adjusting for prognostic features, FOXO6 status was an independent prognostic biomarker of OS and RFS among TNM stage I patients (Table 3). Patients with FOXO6 overexpression had shorter OS (HR = 3.712, 95% CI: 1.225-11.249; *P* = 0.020) and RFS (HR = 3.958, 95% CI: 1.311-11.946; *P* = 0.015) than that with FOXO6 low-expression among TNM stage I patients.

**Table 3:** Univariate and multivariate analysis of FOXO6 associated with survival and recurrence in TNM I gastric cancer patients

Variables*	OS				RFS			
	Univariate		Multivariate		Univariate		Multivariate	
	*P*-value	*P*-value	HR	95% CI	*P*-value	*P*-value	HR	95% CI
Gender (Female vs. Male)	NS	NS			NS	NS		
Age, years (≤ 60 vs. > 60)	NS	NS			NS	NS		
Tumor size (cm) (≤ 5.0 vs. > 5.0)	NS	NS			NS	NS		
Tumor site (Upper vs. Middle/Lower)	NS	NS			NS	NS		
Tumor differentiation (Well/Moderate vs. Poor)	NS	NS			NS	NS		
FOXO6 (Low vs. High)	0.013	0.020	3.712	1.225-11.249	0.008	0.015	3.958	1.311-11.946

Abbreviations: OS overall survival, RFS recurrence-free survival, NS not significant, HR hazard ratio, CI confidential interval.

## DISCUSSION

In mammals, FOXO gene has different expression levels in different tissues. FOXO1 has significant expression in liver tissue and fat tissue, FOXO3 has significant expression in brain tissue, FOXO4 has significant expression in skeletal muscular tissue and FOXO6 has expression in gastric tissue and liver tissue [20, 21]. Although FOXO6 is one of the FOXO family, it differs from other FOXO members in being highly conservative and lacking PKB phosphorylation site of C-terminal [22]. Previous study revealed that FOXO6 overexpression was correlated with oxidative stress, cell proliferation and poor prognosis [20, 23]. However, the prognostic significance of FOXO6 expression in gastric cancer is still unknown. In this study, FOXO6 expression was detected in 192 gastric cancer samples using immunohistochemistry. Our datas showed that FOXO6 expression in gastric cancer samples was higher, compared with para-carcinoma tissues. Moreover, FOXO6 expression was positively correlated with depth of invasion, lymph node metastasis and TNM stage in gastric cancer. In addition, the prognosis analysis showed that the patients with FOXO6 overexpression had poorer survival than that with FOXO6 low-expression. According to the results of multivariate analysis, FOXO6 overexpression was an independent indicator for poor OS and RFS in gastric cancer patients. Furthermore, the prognostic significance of FOXO6 in different risk of subgroups based on tumor size, depth of invasion and lymph node metastasis was assessed, which appeared that FOXO6 could be a negative prognostic biomarker for different risks of gastric cancer patients. Our finding concluded that FOXO6 could serve as a feasible prognostic biomarker of gastric cancer. Several reports found that overexpression of FOXO6 in cancer cells play an important role in tumor progression. Li QY et al [20]. reported that mRNA and protein levels of FOXO6 were upregulated in gastric cancer tissues, FOXO6 overexpression promoted gastric cancer cell proliferation, moreover, FOXO6 induced C-myc expression by associating to HNF4 and mediating histone acetylation, and the dissociation of HDAC3 from the promoter of C-myc gene. Additionally, Chen HY et al. [23] found that FOXO6 was highly expressed in hepatocellular carcinoma sample and was related to oxidative stress levels. Furthermore, FOXO6 expression could be used as a biomarker for deterioration and prognosis of liver cancer. Our results and previous findings powerfully suggest that FOXO6 overexpression may promote tumor progression, and work as an independent predictor for gastric cancer patients.

Extracellular matrix (ECM) degradation is a signal for the beginning of tumor cells invasion and metastasis, and matrix metalloproteases (MMPs) are important molecules involved in degradation of ECM during tumor cells invasion and metastasis [24]. It has been reported that MMP-9 regulate the bioavailability of growth factors and disrupt cell-cell contacts, which could affect cell proliferation and survival [25]. Chu et al. [26] showed that overexpression of MMP-9 was positively correlated with depth of invasion and lymph node metastasis in gastric cancer, and the survival time of patients with MMP-9 overexpression was shorter than that with MMP-9 low-expression. Furthermore, Zhao et al. [27] reported that knockdown of MMP-9 expression could suppress tumor cell growth and invasion of SGC7901 gastric cancer cell *in vitro* and *in vivo*. Our datas found that FOXO6 was positively associated with the expression of MMP-9 in gastric cancer. In addition, FOXO6 overexpression was an important factor of poor prognosis in gastric cancer patients that independent of tumor invasion. In general, FOXO6 overexpression promoting tumor progression indicates that FOXO6 could serve as a potential target in cancer therapy.

The TNM stage is one of the most important factors that affecting the prognosis of gastric cancer patients. However, it is difficult for gastrointestinal surgeon to predict opportunely who would suffer relapse in early-stage patients that have already received radical resection. Many molecular markers have been reported and shown to have potential predictive significance. However, biomarkers which could screen TNM stage I patients with radical resection are still limited. Our results revealed that FOXO6 expression had prognostic significance for OS and RFS in TNM stage I patients. In multivariate analysis, our data reported that FOXO6 was an independent negative prognostic biomarker in TNM stage I patients. These results suggest that FOXO6 expression may serve as a predictive biomarker to identify patients with TNM stage I at high risk of relapse.

In conclusion, this study established a correlation between FOXO6 expression and gastric cancer prognosis. FOXO6 could be a promising predictor for prognosis in gastric cancer. FOXO6 expression could be used to identify high-risk factors of gastric cancer patients, which will help to select appropriate therapies. However, further studies are required to illuminate the potential biological function of FOXO6 in gastric cancer.

## MATERIALS AND METHODS

### Patients and Specimens

The informed consents were provided and experiment was approved by the Institutional Review Board and Human Ethics Committee of Affiliated Cancer Hospital & Institute of Guangzhou Medical University. Informed consents were obtained from all subjects. All methods were performed in accordance with the relevant guidelines and regulations.

All the gastric cancer samples and adjacent non-tumorous gastric tissues were obtained from 192 patients who had received curative resection of gastric cancer between January 2006 and October 2008 at the pathology department, the Affiliated Cancer Hospital & Institute of Guangzhou Medical University (Guangzhou, China). Patient diagnosis was established pathologically, and none of the patients had received chemotherapy or radiotherapy prior to surgery. The cases were selected consecutively on the basis of availability of resection tissues and follow-up data. Relevant clinical pathologic features were all obtained from the patients’ files (Supplementary Table 1). Tumor stage was classified according to the 7th Union International Cancer Control (UICC) TNM staging system [28]. Overall survival (OS) was computed from the date of surgery to the date of death or last follow-up. Recurrence free survival (RFS) was defined as from the date of surgery to the date of relapse, metastasis, or last follow-up.

### Immunohistochemistry staining

A total of 192 gastric cancer tissues and their adjacent non-tumorous tissues were detected by immunohistochemistry (IHC). Formalin-fixed, paraffin-embedded specimens from consenting patients were cut in 4 μm sections. After being baked at 55 °C for 1.5 h, the samples were deparaffinized in xylene and rehydrated using a series of graded alcohols. And then, the tissue slides were treated with 3% hydrogen peroxide in methanol for 10 min to exhaust endogenous peroxidase activity, and the antigens were retrieved in 0.01 M sodium citrate buffer (pH 6.0) using microwave oven, and then preincubated in 10% normal goat serum for 30 min to prevent nonspecific staining. The samples were incubated overnight using a primary antibody, either FOXO6 (Proteintech Group, #19122-1-AP, USA, dilution 1:200) or anti-MMP-9 (Abcam, #ab38898, UK, dilution 1:200), in a humidified container at 4 °C. The tissue slides were treated with a non-biotin horseradish-peroxidase detection system according to the manufacturer's instructions (Gene Tech). Assessments of the staining were scores by two experienced pathologists blinded to the patients’ identity and clinical status. In discrepant cases, a pathologist reviewed the cases and reached the consensus.

Both the extent and intensity of immunostaining were taken into consideration when analyzing the data. The intensity of staining was scored from 0 to 3, and the extent of staining was scored from 0% to 100%. The final quantitation of each staining was obtained by multiplying the two scores. FOXO6 expression was classified as high expression if the score was higher than the median score of 1.1, if the score was 1.1 or less, the case was classified as low expression. MMP-9 expression was considered high if the score was higher than 1.5 (Supplementary Figure 1).

### Follow-up

The follow-up deadline was 30 October 2015. In all the gastric cancer patients (70 females and 122 males), the median follow-up period was 25.5 months, ranging from 5 to 74 months. Patients had follow-up appointments every 1-3 months in the first 3 years, and every 6 months for the next 2 years, and yearly thereafter. Recurrence were confirmed by tumor markers levels including CEA, AFP, CA199, CA125 and CA724, B-type ultrasonic inspection every 3 moths, and computed tomography (CT) or magnetic resonance imaging (MRI) every 6 months after gastrectomy. The main causes of death were gastric cancer recurrence.

### Statistical analysis

All statistical analyses were carried out using SPSS software (version 16.0; Chicago, IL, USA). The chi-square test was used to analyze the correlation of FOXO6 expression with clinical data. The Student's *t*-test was used for comparisons. Correlation of FOXO6 with MMP-9 staining scores was calculated by Pearson χ2 test. Survival curves were generated using the Kaplan-Meier method, and differences between curves were estimated by the log-rank test. The Cox multivariate proportional hazards regression model was used to determine the independent factors that influence prognosis based on the investigated variables. All reported *P* values were two-sided and *P* < 0.05 was considered statistically significant.

## SUPPLEMENTARY MATERIALS FIGURES AND TABLES


