# Supplementary material for: Elevated FOXO6 expression correlates with progression and prognosis in gastric cancer
Source: Oncotarget. 2017 Mar 6;8(19):31682–91. doi: 10.18632/oncotarget.15920 (PMC5458239; doi:10.18632/oncotarget.15920)
Supplement: Supplementary file 1 [file oncotarget-08-31682-s001.pdf]

Elevated FOXO6 expression correlates with progression and prognosis in gastric cancer

SUPPLEMENTARY MATERIALS

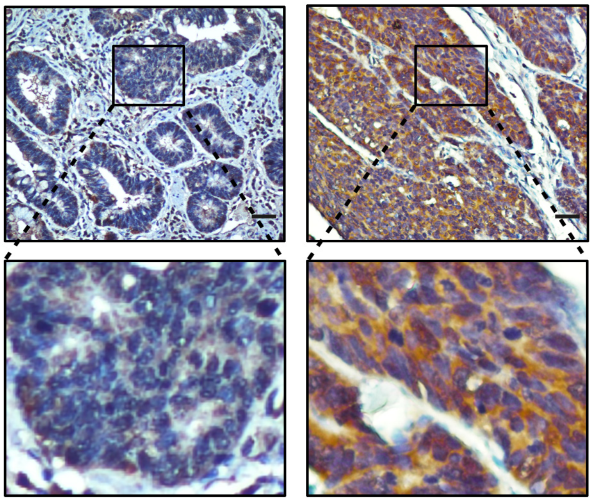

Supplementary Figure 1: MMP-9 was significantly over-expression in gastric cancer. The upper left and right panel represents low and high MMP-9 expression in gastric cancer tissues. Lower panels represent magnified pictures of boxed area in the corresponding upper panels. The scale bar represents 50 μm.

Supplementary Table 1: Patient Characteristics

Variable	No. of patients (%)
No. of patients	192 (100)
Age: Median [range], y	57 [22–82]
Gender	
Female	70 (36.5)
Male	122 (63.5)
Tumor size: Median [range], cm	5.0 [0.5-15.0]
Tumor site	
Upper	81 (42.2)
Middle/Lower	111 (57.8)
Differentiation	
Well	9 (4.7)
Moderate	79 (41.1)
Poor	104 (54.2)
Depth of invasion	
T1	5 (2.6)
T2	69 (35.9)
T3	110 (57.3)
T4	8 (4.2)
Lymph node metastasis	
Negative	52 (27.1)
Positive	140 (72.9)
Stages	
I	48 (25.0)
II	45 (23.4)
III	99 (51.6)
FOXO6 expression	
Low	94 (49.0)
High	98 (51.0)
MMP-9 expression	
Low	100 (52.1)
High	92 (47.9)
Follow-up: Median [range], Months	25.5 [5.0-74.0]
